# Effects of Mycobacteria Major Secretion Protein, Ag85B, on Allergic Inflammation in the Lung

**DOI:** 10.1371/journal.pone.0106807

**Published:** 2014-09-05

**Authors:** Yusuke Tsujimura, Hiroyasu Inada, Misao Yoneda, Tomoyuki Fujita, Kazuhiro Matsuo, Yasuhiro Yasutomi

**Affiliations:** 1 Laboratory of Immunoregulation and Vaccine Research, Tsukuba Primate Research Center, National Institute of Biomedical Innovation, Tsukuba, Ibaraki, Japan; 2 Department of Pharmaceutical Sciences, Suzuka University of Medical Science, Suzuka, Mie, Japan; 3 Department of Pathologic Oncology, Institute of Molecular and Experimental Medicine, Faculty of Medicine, Mie University Graduate School of Medicine, Tsu, Mie, Japan; 4 Research Laboratories, Kyoto R&D Center, Maruho Co., Ltd, Chudoji, Shimogyo-ku, Kyoto, Japan; 5 Research and Development Department, Japan BCG Laboratory, Kiyose, Tokyo, Japan; 6 Department of Immunoregulation, Mie University Graduate School of Medicine, Tsu, Mie, Japan; National Institute of Infectious Diseases, Japan

## Abstract

Many epidemiological studies have suggested that the recent increase in prevalence and severity of allergic diseases such as asthma is inversely correlated with *Mycobacterium bovis* bacillus Calmette Guerin (BCG) vaccination. However, the underlying mechanisms by which mycobacterial components suppress allergic diseases are not yet fully understood. Here we showed the inhibitory mechanisms for development of allergic airway inflammation by using highly purified recombinant Ag85B (rAg85B), which is one of the major protein antigens secreted from *M. tuberculosis*. Ag85B is thought to be a single immunogenic protein that can elicit a strong Th1-type immune response in hosts infected with mycobacteria, including individuals vaccinated with BCG. Administration of rAg85B showed a strong inhibitory effect on the development of allergic airway inflammation with induction of Th1-response and IL-17and IL-22 production. Both cytokines induced by rAg85B were involved in the induction of Th17-related cytokine-production innate immune cells in the lung. Administration of neutralizing antibodies to IL-17 or IL-22 in rAg85B-treated mice revealed that IL-17 induced the infiltration of neutrophils in BAL fluid and that allergen-induced bronchial eosinophilia was inhibited by IL-22. Furthermore, enhancement of the expression of genes associated with tissue homeostasis and wound healing was observed in bronchial tissues after rAg85B administration in a Th17-related cytokine dependent manner. The results of this study provide evidence for the potential usefulness of rAg85B as a novel approach for anti-allergic effect and tissue repair other than the role as a conventional TB vaccine.

## Introduction

Epidemiological studies showed that treatments with bacterial and viral products might be effective therapeutic strategies for suppressing the development of allergic responses [1]–[3]. Administration of mycobacteria, including *Mycobacterium bovis*-Bacillus Calmette Guerin (BCG), has been thought to be effective for preventing the development of asthma by induction of Th1-type immune responses [4], regulatory T (Treg) cells [5], [6] and NKT cells [7], [8]. On the other hand, recent data have revealed that *Mycobacterium tuberculosis* infection induced not only IFN-γ but also IL-17, which promotes granuloma organization followed by neutrophil recruitment, and IL-22, which promotes regeneration and protects against tissue damage [9]. In addition, vaccination with the mycobacteria-secreted immunogenic protein Ag85A had important links with Th1/Th17 cell induction and Treg cell reduction [10]. However, the role of mycobacteria-mediated Th17-related cytokines in allergic asthma remains unknown.

The airway epithelium and innate immune cells are considered to be essential controllers of inflammatory, immune and regenerative responses to allergens that contribute to asthma pathogenesis [11]. Dysfunction of the epithelium leading to chronic injury was suggested to be a consequence of sustained airway inflammation that is associated with Th2-driven adaptive immunity [12]. Tissue homeostasis at exposed surfaces of the lung is regulated by Th17-related cytokines, especially IL-22, in the innate immune system [13]. Therefore, the functional and structural maintenance of tissue might be necessary to induce both innate and adaptive immunity.

One immunogenic protein that can induce a strong Th1-type immune response in hosts sensitized by BCG is thought to be Ag85B. Ag85B is one of the most dominant protein antigens secreted from all mycobacterial species and has been shown to induce substantial Th cell proliferation and vigorous Th1 cytokine production in humans and mice [14]. In addition, we have reported the possibility of using Ag85B DNA as an immunological strategic tool to induce both Th1 and Treg cells in immunotherapy for atopic dermatitis and allergic asthma [15], [16].

In the present study, we found that highly purified recombinant Ag85B protein (rAg85B) had suppressive effects depending on induction of Th1 immune responses in a mouse model of allergic lung inflammation. Remarkably, rAg85B administration also promoted IL-17 and IL-22 production in both Th17 cells in lymph nodes (LNs) and various innate immune cells such as gamma delta T (γδT) cells, NKp46^+^ cells, lymphoid tissue inducer (LTi)-like cells, and CD11c^+^ cells in BAL fluid. More interestingly, Th17-related cytokines induced by rAg85B were involved in enhancement of the expression of genes related to maintenance of tissue homeostasis. This is the first report demonstrating that mycobacteria major secreting protein Ag85B plays an important role in the regulation of allergic airway inflammation by inducing not only a Th1-response but also recruitment of an IL-17 and/or IL-22-producing Th cell subset in LNs and innate immune BAL cells in a manner dependent on Th17-related cytokines in order to retain tissue integrity.

## Materials and Methods

### Animal and Ethic Statement

Specific pathogen-free BALB/c mice (six-week-old, female) were purchased from CLEA Japan. All of the experiments in this study were performed in accordance with the Guidelines for Animal Use and Experimentation, as set out by the National Institute of Biomedical Innovation. The protocol was approved by the Animal Welfare and Animal Care Committee of the National Institute of Biomedical Innovation (Permit Number: DS23-8R2). All animal procedures were used to minimize animal pain and suffering.

### Experimental protocol

BALB/c mice were intraperitoneally immunized with 10 µg ovalbumin (OVA) with 1 mg aluminum hydroxide on days 0 and 14. On days 21 to 25 after the first immunization, mice were exposed to aerosolized 5% OVA for 20 min. Three hours prior to OVA inhalation, the mice were intraperitoneally (i.p.) (100 µg; days 0 and 14) and intranasal ly (i.n.) (20 µg; days 21, 23, and 25) administered rAg85B. OVA-sensitized Balb/c mice were challenged intranasally with PBS, rAg85B, rAg85B plus 5 µg anti-IL-17 Abs and/or 10 µg anti-IL-22 Abs (R&D Systems) with the same time course as that of rAg85B i.n. administration. The isotype-matched control antibody for neutralization experiments was set using normal goat IgG control (R&D systems).

### Recombinant protein Ag85B production

Plasmids containing the Ag85B gene were transformed into *E. coli* TG1. The expressed inclusion body (IB) was harvested from the disrupted cell pellet by a homogenizer with lysis buffer (30 mM sodium phosphaste, 100 mM NaCl, 5 mM EDTA and 0.5% Triton X-100). This IB of Ag85B was unfolded in 8 M urea and refolded by dilution to 0.4 M urea. The urea in the refolding buffer was removed by anion exchange chromatography using 20 mM Tris buffer and 20 mM Tris buffer with 1 M NaCl (pH 8.5). The refolded Ag85B was loaded on a cation exchange column, and crude Ag85B was passed through the resin using 50 mM NaOAc buffer and 50 mM NaOAc buffer with 1 M NaCl (pH 6.0). Finally, Ag85B was purified by anion exchange chromatography using 20 mM Tris buffer and 20 mM Tris buffer with 1 M NaCl (pH 7.6).

### Endotoxin test

The endotoxin value of Ag85B was measured by Kinetic turbidimetric LAL assay kit (Lonza). Test was carried out according to the manufacture's instruction. The endotoxin value was measured kinetically on ELISA after mixing sample and LAL reagent and was calculated automatically according to standard curve. Purified Ag85B had a purity of >95% analyzed by SDS-PAGE and contaminated less than 0.02 EU/mg of endotoxin. Protein quantitation was carried out by UV spectroscopy at 280 nm.

### Isolation and analysis of lymph node and BAL cells

BAL cells were prepared according to a published protocol [16]. Single cell suspensions from BAL fluid and mediastinal lymph nodes (MLNs) were obtained by crushing through cell strainers. Cells were stained with antibodies to the following markers: CD3, CD4, CD8, CD19, CD11b, CD11c, CD25, γδ TCR, NKp46, Gr-1, Siglec-F, CD127, IFN-γ, IL-4, Foxp3, IL-17 and IL-22 (BD). For analysis of intracellular cytokine production, cells were stimulated directly by incubation for 5 h with 50 ng/ml PMA and 750 ng/ml ionomycin (Sigma-Aldrich) at 37°C and with 10 µg/ml brefeldin A (eBioscience) added in the last 3 h. Flow cytometry data collection was performed on a FACS Calibur (BD). Files were analyzed using CellQuest Software (BD).

### Quantification of cytokines and chemokines

Concentrations of cytokines and chemokines in BAL fluid and culture supernatants of OVA-restimulated lymph node cells were determined by ELISA using commercial kits from R&D Systems. Twenty-four hours after the last OVA sensitization, MLNs and BAL fluid were harvested. MLNs were cultured with 50 µg/ml OVA, and cytokines in the culture supernatant were determined 48 h after incubation. The BAL fluid were measured directly.

### Lung histology

The organs were removed and placed in 4% buffered paraformaldehyde (PFA) overnight. Excess paraformaldehyde was removed by incubation in fresh PBS. Fixed tissues were incubated at 4°C in 70% ethanol. PFA-fixed lung sections were stained with hematoxylin and eosin, Masson's trichrome, and α-smooth muscle actin. Peribronchial infiltrates, fibrosis, and smooth muscle hyperplasia were assessed by a semiquantitative score (0–5) by a pathologist.

### Quantitative real-time PCR

RNA was isolated from whole lung tissue using mechanical homogenization and TRIzol reagent (Invitrogen) according to the manufacturer's instructions. RNA concentrations were measured with a Nanodrop ND 1000 (Nucliber). Omniscript reverse tanscriptase was used according to the protocol of the manufacturer (QIAGEN) for the production of cDNA in a reaction volume of 20 ul. Primers for quantitative real-time RT-PCR were designed with the Universal ProbeLibrary Assay Design Center (Roche Applied Science). Reactions were run on an RT-PCR system (LightCycler 480; Roche Applied Science) Samples were normalized to b-actin and displayed as fold induction over naïve or untreated controls unless otherwise stated.

### TLR/NLR ligand screening

The presence of TLR and NLR ligands were tested on recombinant human embryonic kidney 293 (HEK293) cell lines which utilize a nuclear factor-kB inducible SEAP (secreted embryonic alkaline phosphatase) reporter gene as the read-out. These HEK293-derived cells are functionally expressing a given TLR or NOD gene from human or mouse. A recombinant HEK293 cell line for the reporter gene only was used as negative control. Positive control ligands are heat-killed *Listeria monocytogenes* (HKLM) for TLR2, Poly(I:C) for TLR3, Lipopolysaccharide (LPS); K12 for TLR4, Flagellin for TLR5, CL097 for TLR7, CL075 and poly(dT) for TLR8, CpG ODN for TLR9, C12-iEDAP for NOD1, and L18-MDP for NOD2. rAg85B (10 µg/mL) was added to the reaction volume. TLR/NLR ligand screening were performed by InvivoGen.

### Statistical analysis

Data are shown as means±SD. Statistical significance of differences between the OVA-control group and rAg85B-treated group was assessed by the non-parametric Mann-Whitney U-test. Statistical comparisons between groups of rAg85B+isotype control and rAg85B+neutralization antibody were performed using the non-paramatric Kruskal-Wallis H-test.

## Results

### Effects on allergic inflammation by administration of rAg85B

**Figure 1:**
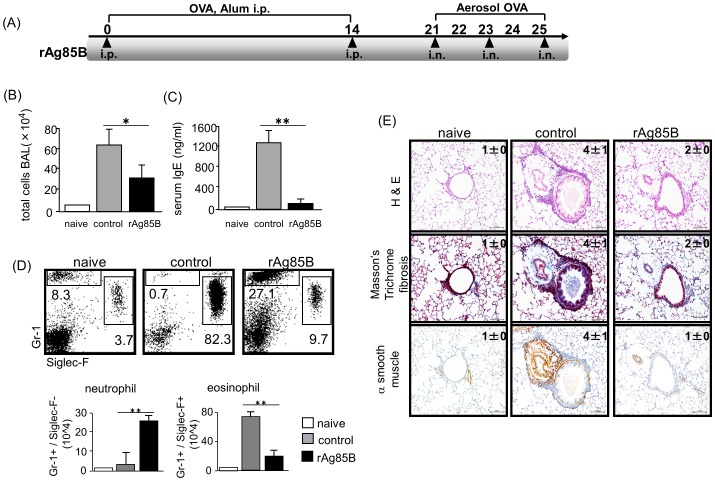
Functions of rAg85B in allergic inflammation. Experimental design used to investigate the effects of rAg85B on OVA-induced allergic lung inflammation (*A*). BALB/c mice were intraperitoneally immunized with OVA on days 0 and 14. On days 21 to 25 after the first immunization, mice were exposed to aerosolized 5% OVA for 20 min. Three hours prior to OVA inhalation, the mice were i.p. (100 µg; days 0 and 14) and i.n. (20 µg; days 21, 23, and 25) administered rAg85B. One day after the last challenge, the BAL cells were counted (*B*) and OVA-specific serum IgE concentrations were determined by ELISA (*C*). Flow cytometry of BAL cells from naïve or OVA sensitized BALB/c mice treated with PBS or rAg85B, stained with anti-Gr-1 and anti-Siglec-F. Numbers adjacent to outlined area indicate percent of eosinophils (Gr-1^dull^, Siglec-F^+^), and neutrophils (Gr-1^+^, Siglec-F^neg^) (*D*). Formalin-fixed tissue sections were stained with hematoxylin and eosin to visualized cell recruitment (upper row, scale bar, 100 mm), Masson's trichrome (center row, scale bar, 100 mm), and α-smooth muscle actin (lower row, scale bar, 50 mm). Numbers in quadrants indicate the score scale from 0 to 5 in each. (*E*). Data are representative of at least three independent experiments. (*P<0.05, **P<0.01 compared with OVA control. error bars, s.d.; n = 6 mice).

To investigate the role of rAg85B in pulmonary allergic inflammation, we examined the frequently used mouse model of ovalbumin (OVA)-induced allergic lung inflammation. The mice were intraperitoneally (i.p.) (days 0 and 14) and intranasally (i.n.) (days 21, 23, and 25) administered with rAg85B (Fig. 1*A*). The purity of rAg85B was evaluated by silver staining of SDS-PAGE gel (Fig. S1) and the Limulus Amebocyte Lysate (LAL) assay (less than 0.02 EU (endotoxin units)/ml). Furthermore rAg85B was not contaminated with any TLR/NLR binding immune stimulants (Table 1). Twenty-four hours after the final OVA challenge, inflammatory cell recruitment into the lungs was analyzed. The OVA-induced allergic manifestation was suppressed with a decrease in the total number of bronchoalveolar lavage (BAL) cells and serum IgE level in the rAg85B-administered mice (Fig. 1*B, 1C*). A marked reduction in eosinophil (Gr-1(+)/Siglec-F(+)) infiltration was observed by flow cytometric (FACS) analysis of BAL in rAg85B-administered mice (Fig. 1*D*). In association with decreased eosinophilia, neutrophil (Gr-1(+)/Siglec-F(-) ) recruitment was seen in rAg85B-administered mice (Fig. 1*D*). These results were confirmed by histopathological observation of hematoxylin and eosin (H&E) staining (Fig. 1*E*). Mice administered rAg85B showed inhibition of infiltration of cells. (Fig. 1*E*). Lung sections were also stained with Masson's trichrome to evaluate fibrosis, and stained with α-smooth muscle actin. Sizes of both the peribronchial smooth muscle area and lung fibrosis area were increased in OVA-sensitized control mice; however, mice administered rAg85B showed strong suppression of both fibrosis and α-smooth muscle actin expression as well as reduction in inflammation severity assessed by H&E staining (Fig. 1*E*). These observations indicated that rAg85B has a critical function of regulating airway inflammation in a mouse model of allergen-induced asthma. Moreover, rAg85B i.n. administration induced wound repair including suppression of both fibrosis and α-smooth muscle actin expression. Incidentally, previous data showed that either i.p. (days 0, 14) or i.n. (days 21, 23, and 25) challenge with rAg85B did not induce strong suppression of Th2-response in OVA-sensitized mice (data not shown).

**Table 1:** Effects of rAg85B to Toll-Like and NOD-Like Receptor.

receptor	No ligand	rAg85B	control (+)
mTLR2	0.1±0.0	0.1±0.0	2.3±0.1
mTLR3	0.1±0.0	0.1±0.0	2.4±0.2
mTLR4	0.1±0.0	0.1±0.0	2.7±0.1
mTLR5	0.1±0.0	0.1±0.0	2.7±0.1
mTLR7	0.1±0.0	0.1±0.0	2.1±0.0
mTLR8	0.1±0.0	0.1±0.0	2.3±0.1
mTLR9	0.1±0.0	0.1±0.0	2.6±0.1
mNOD1	0.1±0.0	0.1±0.0	1.7±0.1
mNOD2	0.2±0.0	0.1±0.0	1.6±0.1

The results are provided as optical density values (650 nm).

The values represent the means and standard deviations of three screenings.

TLR/NLR ligand screening were performed by InvivoGen, as described in Methods.

### Immune deviation from a Th2-response towards a Th1, Th17-related response by rAg85B administration

**Figure 2:**
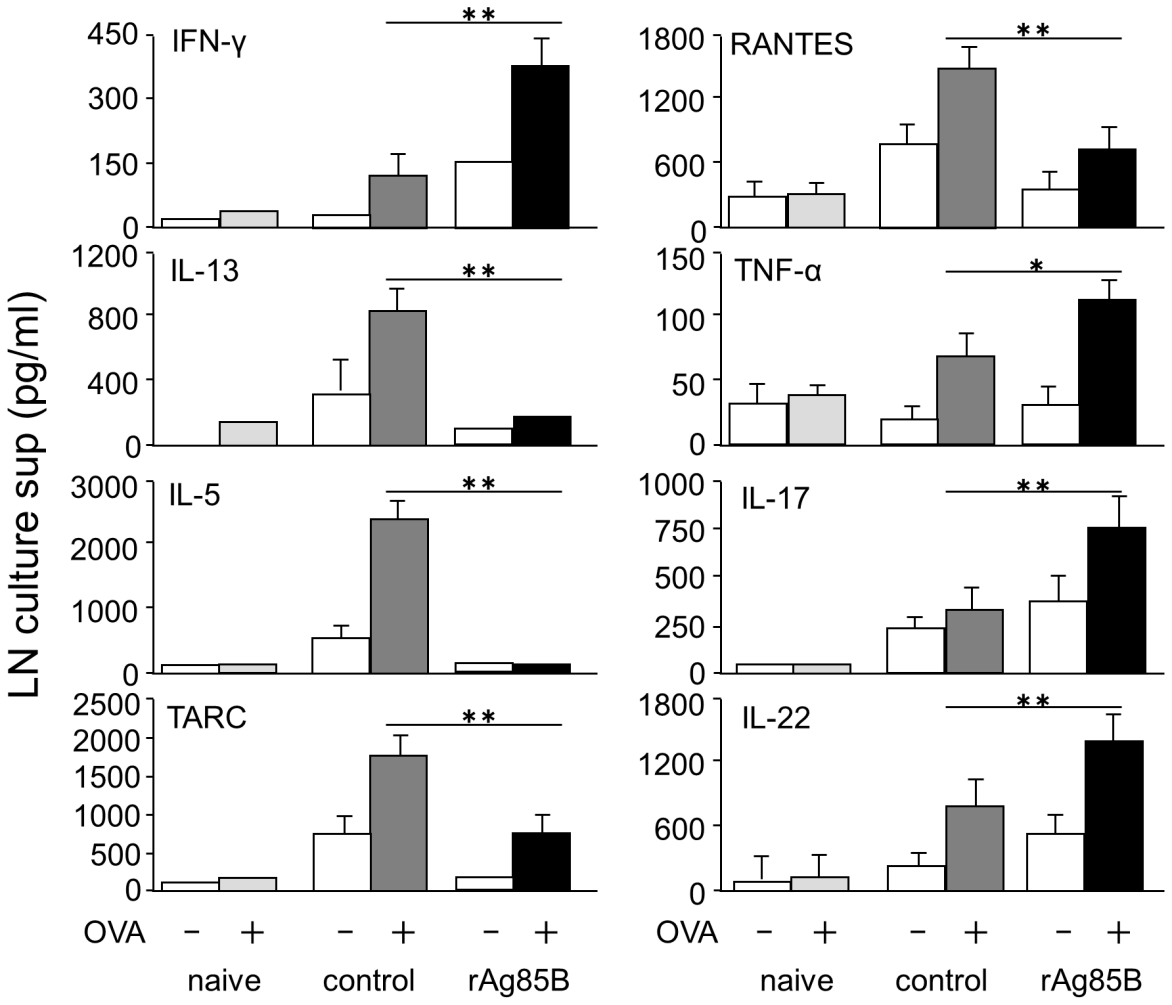
Administaration of rAg85B induced immune deviation from a Th2-response towards a Th1, Th17-related response in OVA-stimulated LN cells. OVA-immunized (i.p., day0 and 14) and sensitized (5% aerosolized-OVA, day21 to 25) BALB/c mice were challenged with PBS or rAg85B protein (i.p. (100 µg; days 0 and 14) and i.n. (20 µg; days 21, 23, and 25)). At 24 h after the last OVA sensitization, mediastinal lymph nodes (MLNs) from naïve or OVA sensitized BALB/c mice treated with PBS or rAg85B, were harvested. MLNs were cultured with OVA (50 µg/ml), and cytokines in the culture supernatant were determined 48 h after incubation by ELISA. Data are representative of at least three independent experiments (*P<0.05, **P<0.01 compared with OVA control. error bars, s.d.; n = 6 mice).

We next assessed the production of OVA-specific cytokines in lymph node cells after *in vitro* stimulation with OVA (Fig. 2). Cells from mediastinal lymph nodes (mLNs) were stimulated *in vitro* with OVA and the production of various types of cytokines was assessed. The level of the Th1 cytokine IFN-γ in culture supernatants of cells from rAg85B-administered mice was increased. On the other hand, the levels of Th2 cytokines IL-5 and IL-13 in culture supernatants of cells from rAg85B-administered mice were lower than those in culture supernatants of cells from control mice. Similarly, mice administered rAg85B showed inhibition of production of the CCL5 (RANTES) and the thymus- and activation-regulated chemokine CCL17 (TARC), which contribute to allergic inflammation. Production of IL-17, IL-22 and TNF-α was also enhanced in culture supernatants of OVA-stimulated mLN cells from rAg85B-administered mice. These results suggested that Th1 and Th17 cytokines are crucial factors in the suppressive effect of rAg85B on airway inflammation.

### CD4^+^ T cells producing IFN-γ and IL-17 were increased in mediastinal lymph nodes by rAg85B administration

**Figure 3:**
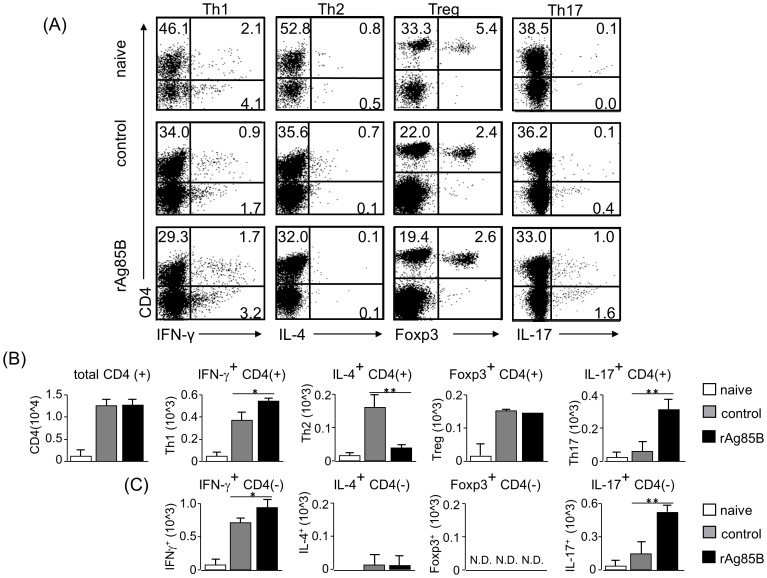
IFN-γ and IL-17-producing CD4 T cell subsets proliferated in lymph nodes after rAg85B administration. OVA-immunized (i.p., day0 and 14) and sensitized (5% aerosolized-OVA, day21 to 25) BALB/c mice were challenged with PBS or rAg85B protein (i.p. (100 µg; days 0 and 14) and i.n. (20 µg; days 21, 23, and 25)). At 24 h after the last OVA sensitization, mediastinal lymph nodes (MLNs) from naïve or OVA sensitized BALB/c mice treated with PBS or rAg85B, were harvested. MLNs were stimulated with ionomycin and PMA for 5 h, and with brefeldin A added in the last 3 h. Flow cytometry of stimulated MLNs from naïve (upper), PBS-treated (middle) and rAg85B protein-treated (lower) OVA-sensitized mice stained with specific antibodies indicated marker. Numbers in quadrants indicate percent of cells in each (*A*). Absolute numbers of various cell populations (above graphs) in lymph nodes (*B*, *C*). Data are representative of three independent experiments (*P<0.05, **P<0.01 compared with OVA control. error bars, s.d.; n = 6 mice).

We next examined Th cell responses in the mouse asthma model by intracellular staining analysis. mLN cells were stimulated with or without PMA and ionomycin, and cell fractions were analyzed by intracellular cytokine staining. Stained CD4^+^ T cells producing IFN-γ or IL-17 were increased in mice administered rAg85B, whereas IL-4-secreting cells were decreased in those mice (Fig. 3*A, 3B*). On the other hand, rAg85B administration was not associated with the induction of Treg cells, which express Foxp3 and CD25, in LNs (Fig. 3*A, 3B*, S2). These results were the same for not only the fraction of CD4^+^ T cells but also the fraction of CD4^−^ cells producing cytokines in mLNs (Fig. 3*C*). These results suggested that rAg85B administration was involved in the induction of IFN-γ or IL-17-producing CD4^+^ T cells and CD4^−^ cells in LNs.

### Mice administered rAg85B showed reduction in levels of Th2 cytokines and chemokines levels and increase in levels of Th1 and Th17 cytokines in BAL

**Figure 4:**
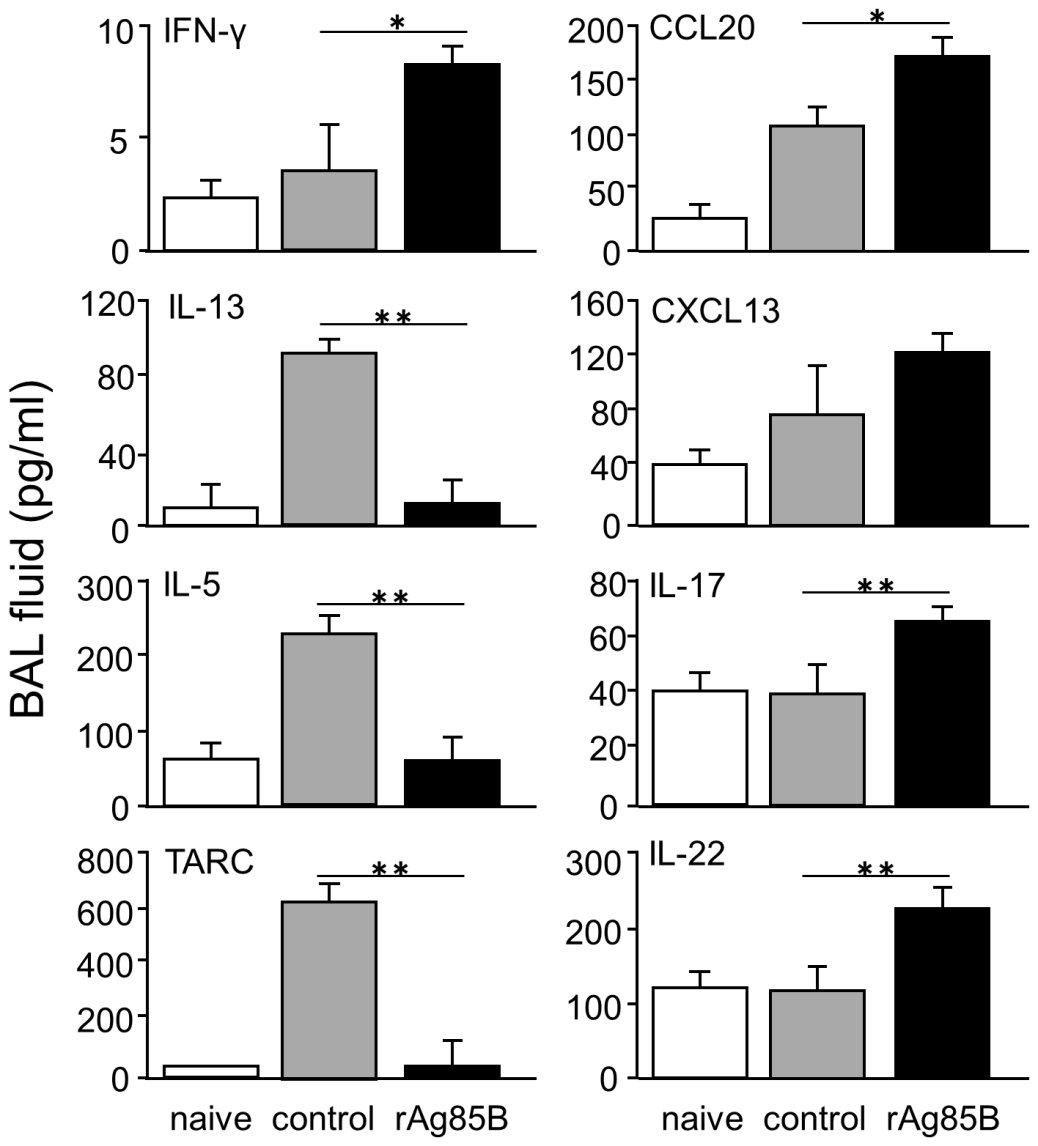
Administration of rAg85B resulted in the reduction of Th2 cytokine and chemokine levels and in the enhancement of Th1 and Th17 cytokine levels in BAL fluid. OVA-immunized (i.p., day0 and 14) and sensitized (5% aerosolized-OVA, day21 to 25) BALB/c mice were challenged with PBS or rAg85B protein (i.p. (100 µg; days 0 and 14) and i.n. (20 µg; days 21, 23, and 25)). At 24 h after the last OVA sensitization, BAL fluid from naïve or OVA sensitized BALB/c mice treated with PBS or rAg85B, were harvested. Levels of cytokines in the BAL fluid were measured directly by ELISA. Data are representative of at least three independent experiments (*P<0.05, **P<0.01 compared with OVA control. error bars, s.d.; n = 6 mice).

The pathogenesis of asthma is associated with many cell types and several molecular/cellular pathways in the lung. Therefore, we investigated whether rAg85B administration regulates various cytokines associated with the pathogenesis of allergic inflammation in BAL fluid. Control mice in which allergic inflammation developed showed increased production of Th2 cytokines and chemokines in BAL fluid, such as IL-13, IL-5 and TARC. Mice administered rAg85B showed inhibition of the induction of IL-13, IL-5 and TARC (Fig. 4). Furthermore, enhancement of IFN-γ, IL-17 and IL-22 production was observed in BAL fluid from mice administered rAg85B. Production of chemokines secreted from non-T cells, CCL20 and CXCL13, was also increased in BAL fluid from rAg85B-administered mice. The chemokine CCL20 is thought to be associated with the recruitment of Th17 lymphocytes and LTi-like or NK-like cells [17], [18], and CXCL13 is a chemokine ligand of C-X-C motif receptor 5 (CXCR5) that is expressed on Lti-like cells. These findings suggested that rAg85B administration was involved in induction of immune responses from both CD4^+^ T cells and other innate cells in BAL fluid of mice in which allergic inflammation has developed.

### rAg85B administration elicits IL-17-producing CD4-negative cells rather than CD4^+^ T cell subsets in BAL fluid

**Figure 5:**
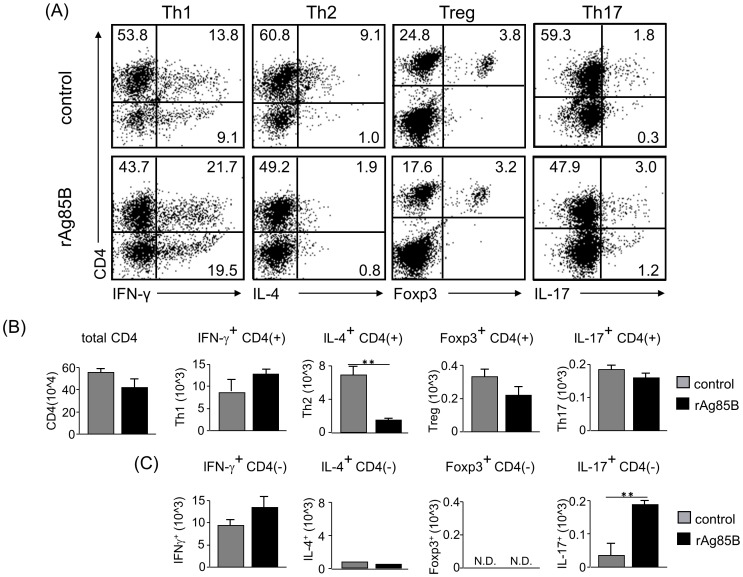
IFN-γ and IL-17-producing CD4-negative cell subsets proliferated in BAL fluid after rAg85B administration. OVA-immunized (i.p., day0 and 14) and sensitized (5% aerosolized-OVA, day21 to 25) BALB/c mice were challenged with PBS or rAg85B protein (i.p. (100 µg; days 0 and 14) and i.n. (20 µg; days 21, 23, and 25)). At 24 h after the last OVA sensitization, BAL fluid from naïve or OVA sensitized BALB/c mice treated with PBS or rAg85B, were harvested. BAL cells were stimulated with ionomycin and PMA for 5 h, and with brefeldin A added in the last 3 h. Flow cytometry of stimulated BAL cells from PBS-treated (upper) and rAg85B protein-treated (lower) OVA-sensitized mice stained with specific antibodies indicated marker. Numbers in quadrants indicate percent of cells in each (*A*). Absolute numbers of various cell populations (above graphs) in BAL fluid (*B*, *C*). Data are representative of three independent experiments (**P<0.01 compared with OVA control. error bars, s.d.; n = 6 mice).

To determine the peripheral Th cell population in the lungs of rAg85B-treated mice, BAL cells from experimental mice were analyzed by intracellular cytokine staining. The percentages of IFN-γ and IL-17-positive cells from rAg85B-administered mice were higher than those from control cells in agreement with the results of FACS analysis of mLN cells (Fig. 5*A*), and Treg cells in BAL fluid from rAg85B-administered mice were also the same as the results for mLN cells (Fig. 5*A*). The absolute number of CD4^+^ T cells stained for IL-17 was not increased in BAL cells from rAg85B-administered mice, unlike the results for mLN cells; however, total IL-17-secreting cells, CD4^−^ IL-17^+^ cells, were increased in rAg85B-administered mice compared with those in control mice (Fig. 5*B, 5C*). CD4^−^ IFN-γ-producing cells were observed in BAL fluid from rAg85B-administered mice as same to CD4^+^ cells. In addition, IL-4-secreting CD4^+^ cells were decreased in rAg85B-treated mice, whereas IL-4-producing CD4^−^ cells were not observed. These observations indicated that IL-17 was produced by CD4^−^ cells rather than by CD4^+^ T cells in BAL, unlike IFN-γ and IL-4. Furthermore, the types of BAL cells greatly changed after rAg85B treatment (Figure S3).

### rAg85B administration was involved in recruitment of innate immune cells that secrete IL-17-related cytokines in BAL fluid

**Figure 6:**
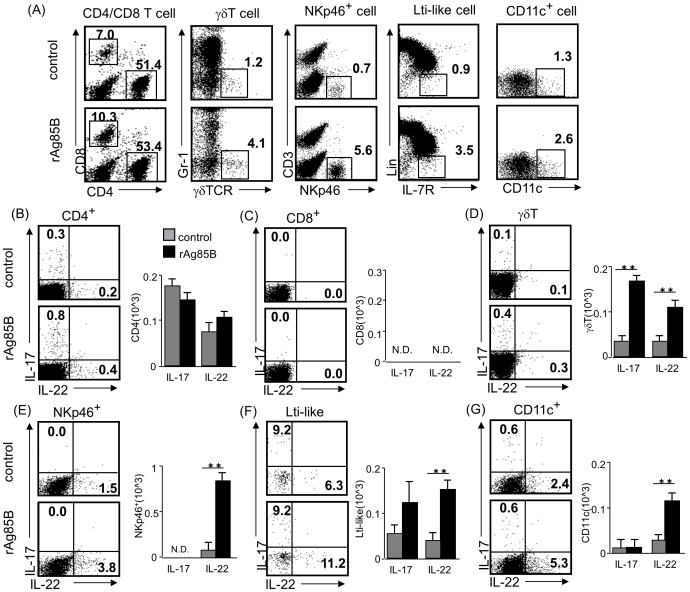
Innate immune cells that secrete Th17-related cytokines are induced by rAg85B administration in BAL fluid. OVA-immunized (i.p., day0 and 14) and sensitized (5% aerosolized-OVA, day21 to 25) BALB/c mice were challenged with PBS or rAg85B protein (i.p. (100 µg; days 0 and 14) and i.n. (20 µg; days 21, 23, and 25)). At 24 h after the last OVA sensitization, BAL fluid from naïve or OVA sensitized BALB/c mice treated with PBS or rAg85B, were harvested. BAL cells were stimulated with ionomycin and PMA for 5 h, and with brefeldin A added in the last 3 h. Flow cytometry of BAL cells from PBS-treated (upper) and rAg85B protein-treated (lower) OVA-sensitized mice stained with anti-CD3, anti-CD4, anti-CD8, anti-Gr-1, anti-γδ TCR, anti-NKp46, anti-CD11c, anti-CD127 (IL-7R) and Lineage specific marker (CD3, CD19, Gr-1, CD11b, CD11c). Numbers in quadrants indicate percent of cells in each (*A*). Intracellular IL-17 and IL-22 staining in indicated cells by flow cytometry (dot plots) and absolute numbers of those cell populations (side graphs) in the BAL fluid (*B, C, D, E, F, G*). Data are representative of at least two independent experiments (**P<0.01 compared with OVA control. error bars, s.d.; n = 6 mice).

Recent studies have demonstrated that IL-17 was not only secreted by Th17 cells and the source of Th17-related cytokines was modified in various environmental conditions [19]. Mice administered rAg85B showed infiltration of CD4-negative immune cells, which secreted IL-17 cytokine in BAL fluid (Fig. 4, Fig. 5). From these findings, we next investigated the proportions of infiltrating CD4^−^ cells that produce IL-17, including γδT cells, IL-7R^+^ Lin^-^ cells (LTi-like cells), CD3^−^ NKp46^+^ cells and CD11c^+^ cells, in BAL fluid from experimental mice. OVA-sensitized BALB/c mice administered rAg85B, but not mice administered PBS, showed an increased number of innate immune cells in BAL fluid (Fig. 6*A*). The percentages of CD4^+^ and CD8^+^ T cells in BAL fluid from rAg85B-administered mice were similar to those in BAL fluid from control mice. However, the percentages of γδT cells, LTi-like cells, NKp46^+^ cells, and CD11c^+^ cells in BAL fluid from rAg85B-administered mice were higher than those in BAL fluid from control mice (Fig. 6*A*). Since innate immune cells, which secrete IL-17 and related cytokines, IL-22, were thought to be induced by rAg85B administration, we next explored the source of IL-17-related cytokines in BAL fluid. Small numbers of IL-17-producing γδT cells, LTi-like cells and CD11c^+^ cells were observed (Fig. 6*D, 6F, 6G*), while production of IL-17 from CD8+ T cells and NKp46^+^ cells was not detected (Fig. 6*C, 6E*). In the present study, a Th17-related cytokine, IL-22, was also detected in BAL fluid from mice administered rAg85B. (Fig. 4). All of the cells from BAL secreting Th17-related cytokines, including CD4^+^ T cells, γδT cells, NKp46^+^ cells, LTi-like cells and CD11c^+^ cells, that were examined in this study showed IL-22 production in mice administered rAg85B (Fig. 6*B, 6D, 6E, 6F, 6G*). On the other hand, production of IL-17 from NKp46^+^ cells and CD11c^+^ cells were not detected (Fig. 6*E, 6G*). Although it is now known that NKT cells, alveolar macrophages and neutrophils might also produce IL-17 in certain conditions, the numbers of these IL-17-secreting cells in BAL fluid from rAg85B-administered mice showed little or no change compared with those in the control group in our experimental setting. Remarkably, these responses induced by rAg85B were observed in allergic animals but not in naïve ones (data not shown).

### Functions of IL-17 and IL-22 in rAg85B-administered mice

**Figure 7:**
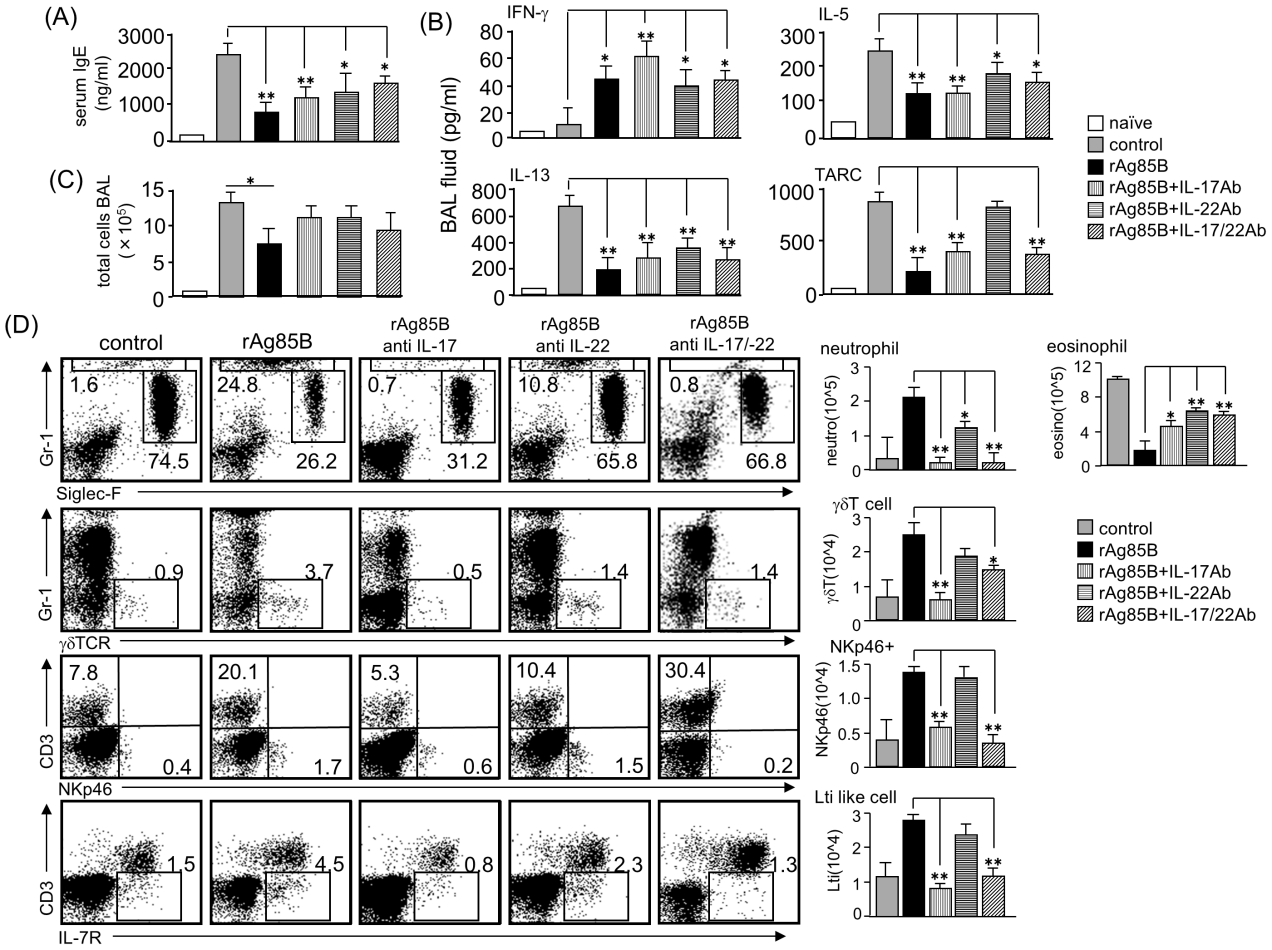
Neutralization of Th17-related cytokines inhibits cell recruitment to the lung but does not change cytokine and chemokine production. OVA-sensitized BALB/c mice (5% aerosolized-OVA, day21 to 25) were challenged intranasally with PBS (control), rAg85B (rAg85B + isotype-matched control antibody (Ab)), rAg85B plus neutralizing IL-17 (rAg85B + IL-17Ab) or IL-22 (rAg85B + IL-22Ab), or a combination of both antibodies (rAg85B +IL-17/22Ab) on days 21, 23, and 25. The isotype control was treated with the same time course as neutralization Ab i.n. administration. One day after the last challenge, OVA-specific serum IgE concentration and levels of cytokines and chemokines in BAL fluid were determined by ELISA (*A*, *B*). BAL cells from naïve or OVA sensitized BALB/c mice treated with PBS or rAg85B with/without neutralization Ab were counted (*C*), and were stained with anti-Gr-1, anti-Siglec-F, anti-gd TCR, anti-CD3, anti-NKp46, and anti-CD127 for flow cytometric analysis. Numbers adjacent to outlined area indicate percent of eosinophils (Gr-1^dull^, Siglec-F^+^), neutrophils (Gr-1^+^, Siglec-F^neg^), γδT cells (Gr-1^neg^, γδTCR^+^), NKp46^+^ cells (CD3^neg^, NKp46^+^), LTi like cells (CD3^neg^, L-7R^+^), and absolute numbers of those cell populations (side graphs) in BAL fluid (*D*). Data are representative of at least two independent experiments (*P<0.05, **P<0.01 compared with rAg85B+isotype control challenged group. error bars, s.d.; n = 6 mice).

We next investigated the importance of Th17-related cytokines by using neutralizing antibodies (Abs) to IL-17 and IL-22 in rAg85B-administered experimental mice. Administration of neutralizing Abs to IL-17 and IL-22 did not show any systemic inhibitory effects induced by rAg85B as a result of IgE production (Fig. 7*A*). Furthermore, neutralization of IL-17 and IL-22 did not restore the functions of rAg85B with immune deviation from a disease-promoting Th2 response towards a Th1 response, whereas inhibition of TARC production regulated by rAg85B was reversed by neutralizing IL-22 Abs treatment (Fig. 7*B*). These results suggested that IL-17 and IL-22 induced by rAg85B have little or no systemic inhibitory effect on the development of allergic inflammation in the lung. Neutralization of IFN-γ at the challenge phase also had little or no suppressive effect on serum IgE expressions and eosinophilia induced by rAg85B treatment. (data not shown). The number of infiltrating cells in BAL fluid were also not changed in mice administered neutralizing Abs to IL-17 and IL-22 (Fig. 7*C*); however, fractions of infiltrating cells in BAL fluid were different. Neutralization of IL-17 by IL-17-specific Abs prevented neutrophil infiltration by rAg85B administration in the airway, and this preventive effect on infiltration of neutrophils was partial in IL-22-specific Abs administered mice (Fig. 7*D*). Eosinophilia suppression by rAg85B administration was reversed by neutralizing IL-22 Abs treatment (Fig. 7*D*). These results parallel previous observations of the specificity of IL-17 and IL-22 effects [20]. Enhancement of innate immune cell recruitment induced by rAg85B was fully reversed by neutralizing IL-17 Abs treatment, and this rAg85B effect was partially reversed by administration of neutralizing IL-22 Abs in γδT cells (Fig. 7*D*). These results showed that Th17-related cytokines induced by rAg85B have pivotal roles in innate immune cell recruitment in BAL fluid and in severity of lung inflammation but not in regulated systemic allergic inflammation involving Th responses.

### Administration of rAg85B promoted Th17-related innate responses in the lung

**Figure 8:**
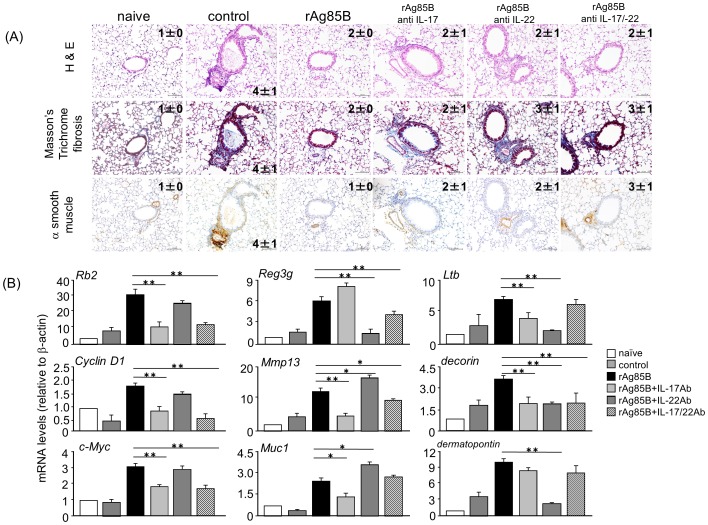
Ag85B administration promotes Th17-related innate responses in the lung. OVA-sensitized BALB/c mice (5% aerosolized-OVA, day21 to 25) were challenged intranasally with PBS (control), rAg85B (rAg85B + isotype control), rAg85B plus neutralizing IL-17 (rAg85B + IL-17Ab) or IL-22 (rAg85B + IL-22Ab), or a combination of both antibodies (rAg85B +IL-17/22Ab) on days 21, 23, and 25. The isotype-matched control antibody was treated with the same time course as neutralization Ab i.n. administration. Lungs from naïve or OVA sensitized BALB/c mice treated with PBS or rAg85B with/without neutralization Ab were sampled one day after the last challenge for histological analysis and quantification of mRNA levels. Lung sections were stained with hematoxylin and eosin (left row, scale bar, 100 mm), Masson's trichrome (center row, scale bar, 100 mm), α-smooth muscle actin (right row, scale bar, 50 mm). Numbers in quadrants indicate the score scale from 0 to 5 in each (*A*). Real-time RT-PCR was performed for the indicated molecules expression on RNA isolated from individual mice lungs (*B*). Data are representative of at least two independent experiments (*P<0.05, **P<0.01 compared with rAg85B+isotype control challenged group. error bars, s.d.; n = 6 mice).

**Figure 9:**
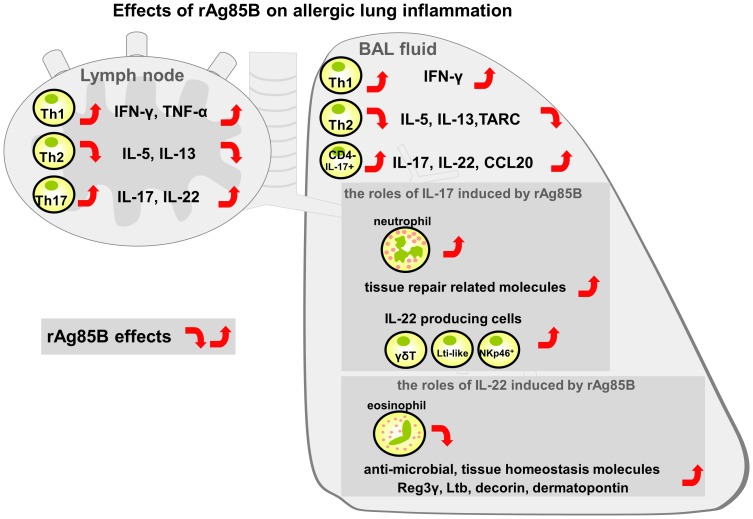
Schematic illustration of the proposed effects of rAg85B in a mouse model of allergic inflammation. IFN-γ and IL-17-producing Th cells are induced in regional lymph nodes by rAg85B challenge, however, Th17 cells do not enter the lung unlike Th1 cells. Th17-related cytokine-secreting cells in lungs from rAg85B-administered mice are innate immune cells including γδT cells, IL-7R^+^ Lin^−^ cells, CD3^−^ NKp46^+^ cells and CD11c^+^ cells. IL-17 and IL-22 induced by rAg85B in an allergic environment have crucial roles in not only anti-allergic effects but also regulation of tissue homeostatic reactions.

Our data suggested an important link between rAg85B and airway innate immune cells producing IL-17 and IL-22 that contributed to the homeostasis expression of Th17-related cytokine response genes. However, these two cytokines induced by rAg85B administration did not clearly show inhibitory effects on systemic allergy responses (Fig. 7*A, 7B*). From these findings, we next explored the relationship between the roles of airway innate immune cells and wound repair in mice that received i.n. administration of rAg85B. Mice that received IL-17 or IL-22 or both neutralizing antibodies showed a marked induction of fibrosis and actin staining but incomplete cancellation of rAg85B suppressive effects at the same levels as those in OVA control mice (Fig. 8*A*). Histological findings suggested that IL-17 and IL-22 induced by rAg85B i.n. administration were partially involved in regulation of local tissue allergic inflammation. The inhibition of rAg85B effects by neutralizing Abs of IL-17 and IL-22 to allergic inflammation was partial; however, tissue repair in lungs was seen in rAg85B-administered mice by histopathological examination. These results led us to hypothesize that IL-17 and IL-22 induced by rAg85B induced local tissue remodeling/repair molecules. To confirm this, the induction of tissue homeostasis-related gene expression in rAg85B-administered mice was examined by real-time RT-PCR. Rb2, Cyclin D1 and c-Myc are associated with wound healing, tissue repair and remodeling including proliferative molecules [21]. Muc1 [22], [23], matrix metalloproteinase 13 (MMP13) [24], and the extracellular matrix proteins decorin and dermatopontin [25] produce protective mucus. Lymphotoxin-beta (Ltb) is a molecule related to signaling in stromal cells to produce factors that organize lymphoid cells into lymph nodes [26]. The transcription of Reg3γ is involved in tissue repair and antimicrobial responses [27]. The expression of these genes involved in innate immune response-mediated signaling was significantly enhanced in the lungs of rAg85B-administered mice (Fig. 8*B*). The increases in mRNA levels of all molecules other than Reg3γ and dermatopontin were inhibited by treatment with neutralizing Abs of IL-17 (Fig. 8*B*). On the other hand, the expression of mRNA of molecules enhanced by rAg85B administration was decreased after treatment with IL-22 neutralizing Abs except for Rb2, Cyclin D1, c-Myc, Mmp13 and Muc1 (Fig. 8*B*). These results suggested that IL-17 and IL-22 induced by rAg85B administration affected induction of pulmonary innate response. In conclusion, IL-17 induced by rAg85B administration induced the expression of various types of wound healing, tissue repair and remodeling molecules. Interestingly, IL-22 in rAg85B-immunized mice induces the expression of molecules mainly associated antimicrobial responses such as Reg3γ, decorin, dermatopontin and Ltb. In summary, Th1 and Th17 cells are induced in regional lymph nodes by administration of rAg85B; however, Th17 cells are not induced in BAL unlike in Th1 cells. IL-17 is produced by innate immune cells with IL-22 production. IL-17 and IL-22 are important in not only anti-allergic effects, such as eosinophil inhibition, but also wound healing and tissue repair in the lung (Fig. 9).

## Discussion

Results of several experimental studies on mycobacteria involving mycobacterial antigens in mouse models of allergic airway inflammation have been reported. In murine asthma models, intranasal administration of BCG suppressed asthma manifestations probably through Th1 response [2], [4], Treg cells [5], [6], [28], or NKT cells [7], [8], [29]. In our experimental setting using rAg85B protein, we did not find any detectable effect or substantial change in numbers of both NKT cells and Treg cells in BAL fluid from rAg85B-administered mice. Moreover, microarray analysis revealed that the gene expression pattern of splenocytes stimulated with rAg85B and that of splenocytes stimulated with BCG were very different (data not shown). This discrepancy in the effects of vaccination with BCG and vaccination with rAg85B might be related to the factors affecting immune responses. BCG contains many essential components to induce early immune response such as glycolipid and DNA, whereas rAg85B is a single immunogenic protein.

The present study indicated that Th17-related immune responses induced by rAg85B administration had a suppressive effect on allergic airway inflammation, and we attributed this suppressive effect to the larger proportion of Th17-related cytokine-producing innate immune cells in BAL fluid. It has been reported that Mycobacterium antigens increased the number of γδT cells that express IFN-γ [30] or IL-17 [31], and these responses induce healing to epithelial surfaces [32]. Given the integral role of γδT cells in innate immunity, γδT cells are one of the crucial factors in the rAg85B immune regulatory functions. Interestingly, our study also showed that IL-22-producing cells in lungs of rAg85B-administered mice were NKp46^+^ cells, LTi-like cells, γδT cells and CD11c^+^ cells. This is the first time demonstration of an important link between the mycobacterium antigen rAg85B and IL-22-producing cells. Although we could not rule out the possibility of Th17 cytokine-producing cells other than those described here, NKp46^+^ cells, LTi-like cells, γδT cells and CD11c^+^ cells were thought to be rapid innate sources of IL-22, which is required in the early stage to maintain epithelial cell integrity and to suppress eosinophilia. Moreover, IL-22 can act synergistically or additively with other cytokines, including IL-17 or TNF, to promote gene expression for antimicrobial peptides, chemokines, matrix metalloproteinases, cytokines, and acute-phase proteins from epithelial cells in the lung [33]. These findings also support our results showing that simultaneous induction of these cytokines and expression of many genes may be beneficial functions of rAg85B treatment in local allergic pathology.

Pulmonary infection of mycobacteria induced not only a neutrophil-mediated response but also T cell-mediated IFN-γ production and granuloma formation depending on IL-17 from especially γδT cells [34]. The hallmark of mycobacterial infection in the lung is granuloma formation with infiltrating neutrophils, which creates an immune microenvironment in which the infection can be controlled. On the other hand, it also provides the mycobacterium with a niche in which it can survive, modulating the immune response to ensure its survival without damage over a long period of time [35], [36]. Mature granulomas include fibroblasts and extracellular matrix, which surround and separate the granulomas from the normal environment. Administration of anti-IL-17 Abs during the inhalatory challenge phase abolished the bronchial neutrophilia and the upregulation of genes related to tissue repair and homeostasis observed in rAg85B-administered mice in the present study. Neutrophils may also promote epithelial healing [37] and are now known to be rich sources of prestored and expressible proteins [38] that may directly promote wound healing [39], [40]. In the present study, induction of neutrophilia and upregulation of described genes related to wound healing with suppression of tissue injury might be the mechanisms of granuloma formulation induced by mycobacteria infection.

In the present study, IFN-γ and Th17-related cytokines were key factors to regulate allergic severity in our experimental setting. Although infiltration of Th1 and Th17 cells elicited by rAg85B was induced in pulmonary lymph nodes, such effector cells were not increased in BAL fluid of mice showing anti-allergic effects of Ag85B administration. Moreover, our results suggested that the accumulation of neutrophils and IL-17 and/or IL-22-producing innate immune cells contributed to the homeostatic functions in the Th1-balanced environment induced by rAg85B administration. These findings provide a new insight into the regulatory effects of various innate immune factors induced by the mycobacteria major secretion protein rAg85B in allergic inflammation.

## Supporting Information

Figure S1 **SDS-PAGE separation and silver staining of rAg85B.** The recombinant purified Ag85B was solubilized in sample buffer to the desired concentration, and boiled for 5 min. 15 µl/well from each samples were separated on 10% SDS gel using mini-PROTEAN electrophoresis instrument (Bio-Rad Laboratories). Silver staining of the gel was performed according to the standard protocol of EzStain Silver (ATTO). Various concentration of rAg85B on the gel (12.5, 25, 50, 100, 200, and 400 ng).
(TIF)

Figure S2 **CD4^+^ Foxp3^+^ T cells were almost expressing CD25.** OVA-immunized (i.p., day0 and 14) and sensitized (5% aerosolized-OVA, day21 to 25) BALB/c mice were challenged with PBS or rAg85B protein (i.p. (100 µg; days 0 and 14) and i.n. (20 µg; days 21, 23, and 25)). At 24 h after the last OVA sensitization, mediastinal lymph nodes (MLNs) from naïve or OVA sensitized BALB/c mice treated with PBS or rAg85B, were harvested. MLNs cells from naïve (upper), PBS-treated (middle) and rAg85B protein-treated (lower) OVA-sensitized mice were stained with anti-CD4 and anti-Foxp3. Cells in R1-R4 were analyzed for the expression of CD25.
(TIF)

Figure S3 **The composition of BAL cells in rAg85B administered mice.** OVA-immunized (i.p., day0 and 14) and sensitized (5% aerosolized-OVA, day21 to 25) BALB/c mice were challenged with PBS (control) or rAg85B (i.p. (100 µg; days 0 and 14) and i.n. (20 µg; days 21, 23, and 25)). One day after the last challenge, BAL cells from OVA sensitized BALB/c mice treated with PBS or rAg85B were counted. Different BAL cells populations were measured by surface staining. Flow cytometry of BAL cells stained with anti-CD3, anti-CD4, anti-CD8, anti-Gr-1, anti-γδ TCR, anti-NKp46, anti-CD11c, anti-CD127 (IL-7R) and Lineage specific marker (CD3, CD19, Gr-1, CD11b, CD11c).
(TIF)
