# Supplementary figures and images for: Effects of Mycobacteria Major Secretion Protein, Ag85B, on Allergic Inflammation in the Lung
Source: PLoS One. 2014 Sep 5;9(9):e106807. doi: 10.1371/journal.pone.0106807 (PMC4156387; doi:10.1371/journal.pone.0106807)

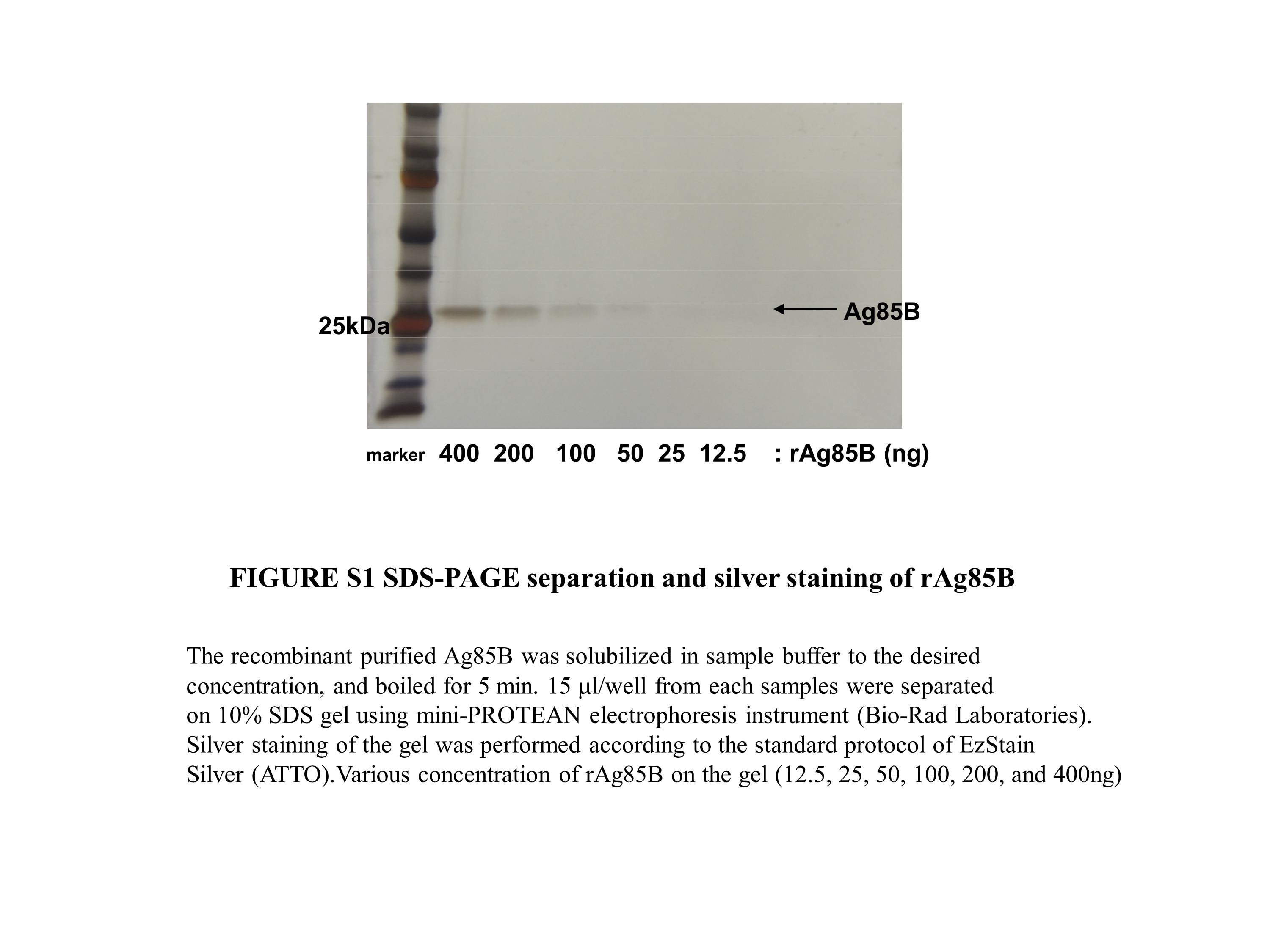

Supplement: Figure S1 — SDS-PAGE separation and silver staining of rAg85B. The recombinant purified Ag85B was solubilized in sample buffer to the desired concentration, and boiled for 5 min. 15 µl/well from each samples were separated on 10% SDS gel using mini-PROTEAN electrophoresis instrument (Bio-Rad Laboratories). Silver staining of the gel was performed according to the standard protocol of EzStain Silver (ATTO). Various concentration of rAg85B on the gel (12.5, 25, 50, 100, 200, and 400 ng). (TIF) [file pone.0106807.s001.tif]

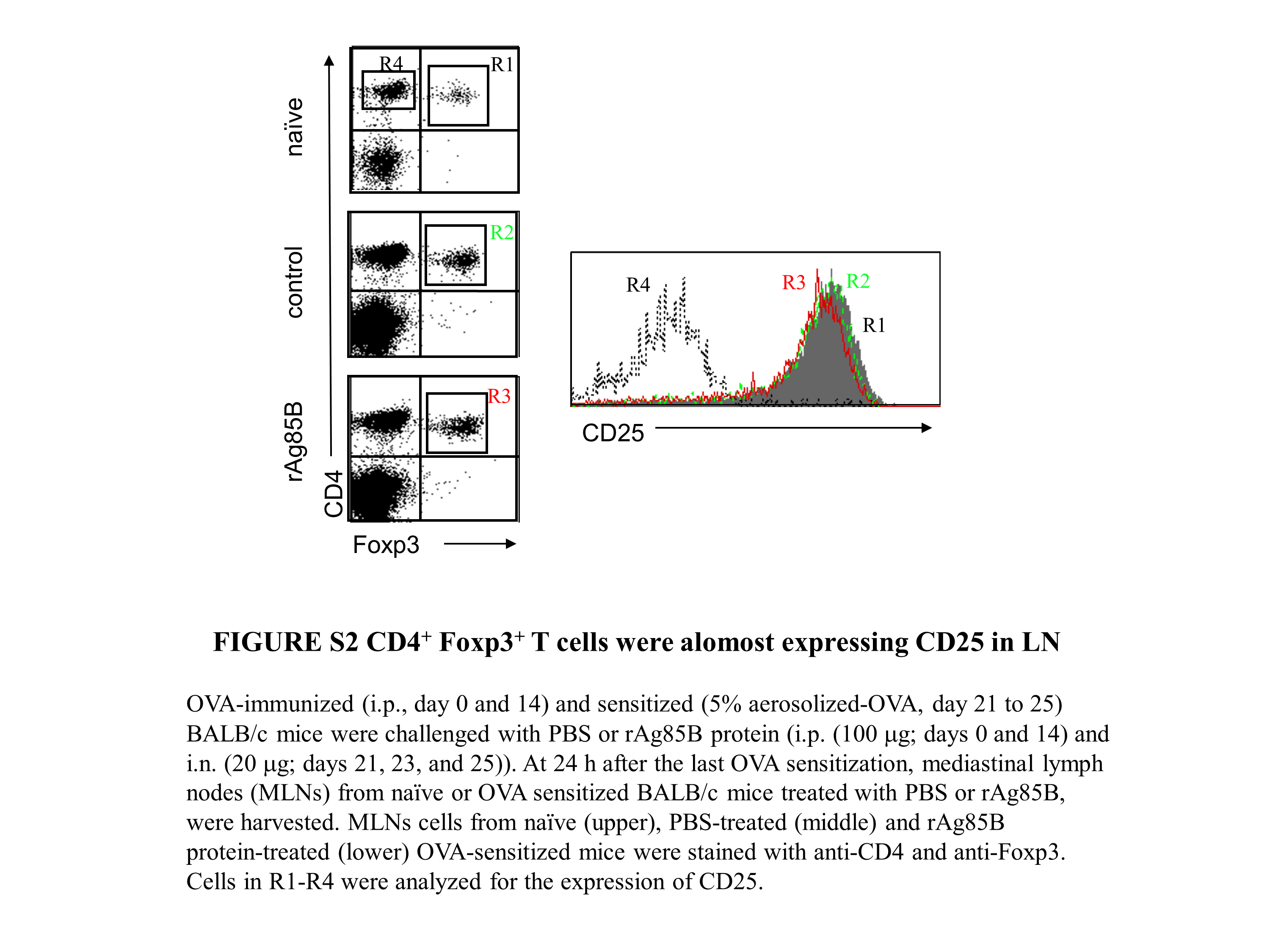

Supplement: Figure S2 — CD4+ Foxp3+ T cells were almost expressing CD25. OVA-immunized (i.p., day0 and 14) and sensitized (5% aerosolized-OVA, day21 to 25) BALB/c mice were challenged with PBS or rAg85B protein (i.p. (100 µg; days 0 and 14) and i.n. (20 µg; days 21, 23, and 25)). At 24 h after the last OVA sensitization, mediastinal lymph nodes (MLNs) from naïve or OVA sensitized BALB/c mice treated with PBS or rAg85B, were harvested. MLNs cells from naïve (upper), PBS-treated (middle) and rAg85B protein-treated (lower) OVA-sensitized mice were stained with anti-CD4 and anti-Foxp3. Cells in R1-R4 were analyzed for the expression of CD25. (TIF) [file pone.0106807.s002.tif]

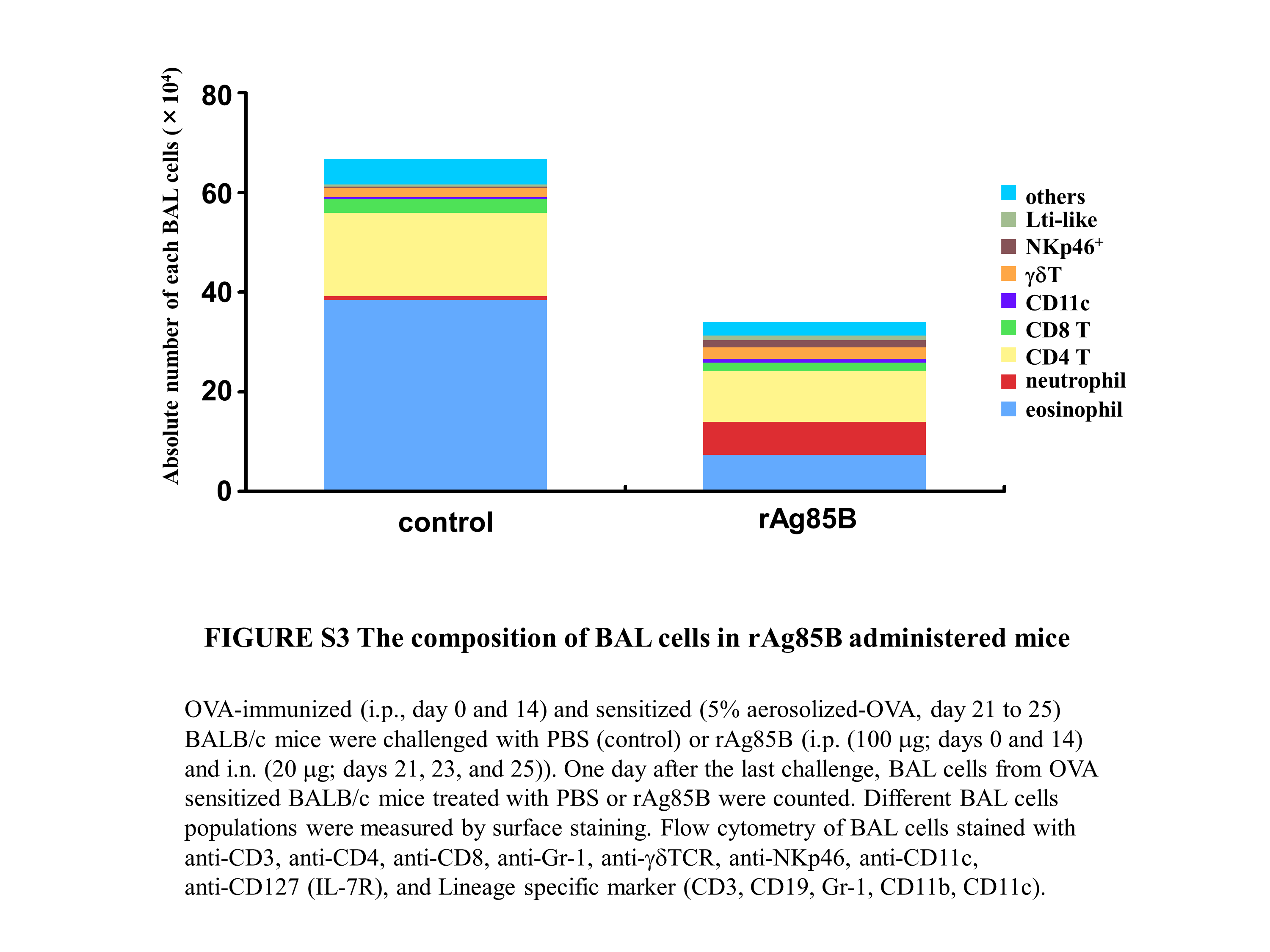

Supplement: Figure S3 — The composition of BAL cells in rAg85B administered mice. OVA-immunized (i.p., day0 and 14) and sensitized (5% aerosolized-OVA, day21 to 25) BALB/c mice were challenged with PBS (control) or rAg85B (i.p. (100 µg; days 0 and 14) and i.n. (20 µg; days 21, 23, and 25)). One day after the last challenge, BAL cells from OVA sensitized BALB/c mice treated with PBS or rAg85B were counted. Different BAL cells populations were measured by surface staining. Flow cytometry of BAL cells stained with anti-CD3, anti-CD4, anti-CD8, anti-Gr-1, anti-γδ TCR, anti-NKp46, anti-CD11c, anti-CD127 (IL-7R) and Lineage specific marker (CD3, CD19, Gr-1, CD11b, CD11c). (TIF) [file pone.0106807.s003.tif]
